# Climate change and *Vibrio vulnificus* dynamics: A blueprint for infectious diseases

**DOI:** 10.1371/journal.ppat.1012767

**Published:** 2024-12-16

**Authors:** Jane M. Jayakumar, Jaime Martinez-Urtaza, Kyle D. Brumfield, Antarpreet S. Jutla, Rita R. Colwell, Otto X. Cordero, Salvador Almagro-Moreno

**Affiliations:** 1 Burnett School of Biomedical Sciences, University of Central Florida, Orlando Florida, United States of America; 2 Department de Genetica I de Microbiologia, Facultat de Biociencies, Universitat Autonoma de Barcelona, Barcelona Spain; 3 University of Maryland Institute for Advanced Computer Studies, University of Maryland, College Park Maryland United States of America; 4 Department of Cell Biology and Molecular Genetics, University of Maryland, College Park, Maryland United States of America; 5 Geohealth and Hydrology Laboratory, Department of Environmental engineering Sciences, University of Florida, Gainesville Florida United States of America; 6 Johns Hopkins Bloomberg School of Public Health, Baltimore Maryland United States of America; 7 Department of Civil and Environmental Engineering, Massachusetts Institute of Technology, Cambridge Maryland United States of America; Monash University, UNITED STATES OF AMERICA

## Abstract

Climate change is having increasingly profound effects on human health, notably those associated with the occurrence, distribution, and transmission of infectious diseases. The number of disparate ecological parameters and pathogens affected by climate change are vast and expansive. Disentangling the complex relationship between these variables is critical for the development of effective countermeasures against its effects. The pathogen *Vibrio vulnificus*, a naturally occurring aquatic bacterium that causes fulminant septicemia, represents a quintessential climate-sensitive organism. In this review, we use *V*. *vulnificus* as a model organism to elucidate the intricate network of interactions between climatic factors and pathogens, with the objective of identifying common patterns by which climate change is affecting their disease burden. Recent findings indicate that in regions native to *V*. *vulnificus* or related pathogens, climate-driven natural disasters are the chief contributors to their disease outbreaks. Concurrently, climate change is increasing the environmental suitability of areas non-endemic to their diseases, promoting a surge in their natural populations and transmission dynamics, thus elevating the risk of new outbreaks. We highlight potential risk factors and climatic drivers aggravating the threat of *V*. *vulnificus* transmission under both scenarios and propose potential measures for mitigating its impact. By defining the mechanisms by which climate change influences *V*. *vulnificus* disease burden, we aim to shed light on the transmission dynamics of related disease-causing agents, thereby laying the groundwork for early warning systems and broadly applicable control measures.

## Introduction

Climate change, fueled by anthropogenic factors, represents one of the greatest threats to human health in the 21st century [1,2]. Among the numerous health crises driven by climate change, the emergence and geographical expansion of pathogens and their diseases represents a critical concern [3,4]. To date, climate change has resulted in the occurrence or aggravation of more than 58% of all infectious diseases [5]. For instance, altered weather patterns have increased the spread of vectors like mosquitos and ticks that carry diseases such as dengue and Lyme disease [6–8]. Furthermore, rising temperatures have prolonged summer periods, amplifying incidences of food- and water-borne diseases like those caused by *Vibrio* spp., or *Cryptosporidium* spp. [6,9]. Such temperature anomalies have also fueled natural disasters, like heatwaves and hurricanes, afflicting communities with outbreaks of pathogens such as *Clostridium tetani*, *Bacillus anthracis*, or *Leptospira* spp. [9–12]. In addition, habitat disruption due to climatic disasters or anthropogenic activities has resulted in unprecedented biodiversity loss, forcing contact between wildlife and humans, and causing a spillover of zoonotic pathogens like Ebola virus [5,6]. Moreover, increased air pollution due to urbanization and disasters like forest fires have increased human susceptibility and exposure to respiratory pathogens like the influenza virus [13]. These examples represent a fraction of the current and future health threats that arise during the unfolding of this global climatic crisis. Moreover, they underline the synergistic and often indirect mechanisms by which various climatic factors drive the emergence and spread of infectious diseases. By identifying factors driving the occurrence and transmission of disease-causing agents, early warning systems can be developed for public health intervention in high-risk areas [14,15]. However, at present, actions against climate change remain in their infancy and are often limited to developed countries, whereas its effects are disproportionately severe in low-income nations [2]. This disparity necessitates the development and implementation of strategies that address the impact of climate change on the emergence and dissemination of infectious diseases at a global scale [2,7].

**Fig 1:**
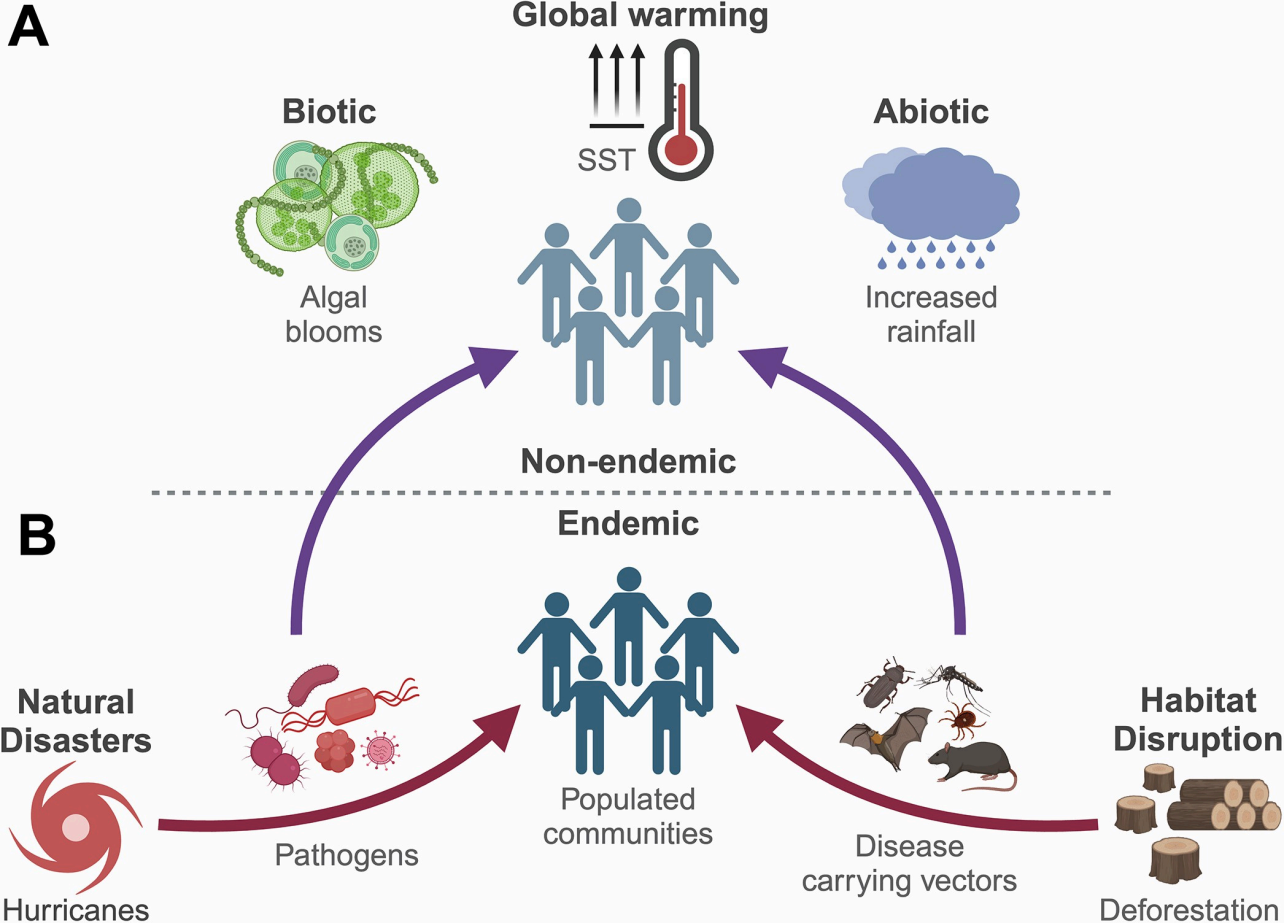
Influence of climate change on infectious disease burden. (**A**) Non-endemic. Climate change driven alterations in biotic, abiotic, or weather patterns improves the ecological suitability of non-endemic areas resulting in increased proliferation of pathogens or their vectors and the spatiotemporal expansion of their diseases. (**B**) Endemic. In regions native to pathogens or their vectors, natural disasters or habitat disruptions force their increased interaction with hosts, particularly in densely populated areas, resulting in severe disease outbreaks. Arrows indicate direction of disease transmission. Created in BioRender. Almagro-Moreno, S. (2024) https://BioRender.com/j65b451.

As highlighted above, climate change influences infectious disease dynamics via numerous unique and nonlinear mechanisms and affects a wide variety of pathogens [5]. Disentangling the complex network of interactions between and among these factors and infectious agents is vital to identify common trends useful for the development of surveillance systems and broadly applicable control measures. A careful look at epidemiological patterns [5–12] suggests that most climatic pathways driving disease transmission can be grouped into 2 categories: those affecting regions “endemic” or “non-endemic” to infectious diseases (**Fig 1**). *Endemic* describes regions in which conditions are favorable for the proliferation of pathogenic agents native to that region, and where their disease incidences are common. Per contra, *non-endemic* regions are those where pathogens are either absent or present in very low or undetectable numbers, but where conditions are typically unfavorable for their proliferation or disease transmission. Emerging trends indicate that in endemic regions, climate-driven extreme weather events or habitat disruptions force the increased interaction between pathogens and their hosts, either by direct contact or mediated by vectors, leading to the emergence of severe disease outbreaks [5,6,9–12]. In non-endemic regions, changes in climatic factors, such as temperature and rainfall patterns, are improving the ecological suitability for proliferation of such pathogens or their vectors [6–9]. As a result, these pathogens are increasing in densities and transmission potential geographically, threatening immunologically naïve populations in such areas. A prime example of pathogens observing both these climatic trends are the non-cholera *Vibrio* spp., which are autochthonous to the aquatic environment [16–20]. These *Vibrio* spp. are undergoing population increases poleward accompanied by associated disease outbreaks along coastal communities [16–20]. Given their high growth rate and rapid response to climate-driven changes in the environment, non-cholera *Vibrio* spp. represent valuable microbial indicators of climate change [19,21]. Among these *Vibrio* spp., *Vibrio vulnificus* stands out as a quintessential model of a climate-sensitive pathogen that follows these observed patterns [17,22,23].

*V*. *vulnificus* is a natural inhabitant of estuaries and proliferates when temperatures are greater than 18°C and salinities are between 10 and 20 parts per thousand (ppt) [24,25]. It is transmitted to humans by the ingestion of seafood or contact of open wounds with water harboring the bacterium, causing primary septicemia for which the mortality rate exceeds 50% [26–29]. *V*. *vulnificus* is the leading cause of non-cholera *Vibrio*-related deaths globally and has one of the fastest rates of disease progression [30–32]. *V*. *vulnificus* is also the costliest marine-borne pathogen with an estimated economic burden of $267 million annually [33]. *V*. *vulnificus* accounts for nearly one-third of the total seafood borne disease costs in the United States [33]. *V*. *vulnificus* infections commonly occur during summer months when temperatures are elevated, promoting growth of the bacterium, and when water-based outdoor recreation is at its peak [19]. Alarmingly, in recent years, *V*. *vulnificus* cases have been increasing rapidly in areas where its infections were previously uncommon [17,34]. For instance, a recent investigation of pathogenic *Vibrio* spp. in Chesapeake Bay identified an extended seasonality of *V*. *vulnificus* growth during the fall months [35]. Subsequently, an increase in the number of *V*. *vulnificus* infections in Maryland was reported [36]. Similarly, locations where *V*. *vulnificus* diseases were extremely rare a decade ago, e.g., Connecticut, New York, or Rhode Island, reported fatalities in 2023 and 2024 correlated with warming temperatures [37,38]. Moreover, climatic disasters like hurricanes and heatwaves have caused large-scale outbreaks of *V*. *vulnificus* worldwide. Examples include outbreaks in Florida following Hurricanes Ian and Helene in 2022 and 2024, respectively [22,39] and those after extreme heatwaves in Sweden and Finland in 2006 and 2014, respectively [20,34]. Nonetheless, *V*. *vulnificus*, like other pathogenic *Vibrio* spp., plays an important role in the biogeochemistry of the planet, and hence, cannot be eradicated. For instance, the bacterium forms commensal relationships with crustaceans and zooplankton, contributing to the degradation of polymeric substances, such as chitin, and aiding in carbon and nitrogen cycling [40]. This underscores the complex relationship between *V*. *vulnificus* and its aquatic ecosystem and necessitates the development of alternative strategies to counteract its disease outcomes.

Given the sensitivity of *V*. *vulnificus* to the consequences of global warming, notably natural disasters and alterations in ecological factors, the bacterium is often considered a “microbial barometer of climate change” [21]. This makes *V*. *vulnificus* an ideal candidate to study the impact of climate change on disease dynamics at endemic and non-endemic levels and to identify common climatic patterns driving disease emergence and spread. Therefore, in this review we summarize current trends that fuel *V*. *vulnificus* outbreaks in endemic regions and create conditions favorable for its transmission in regions non-endemic to its disease. We further highlight key risk factors that threaten communities in disaster-prone regions and increase the suitability of previously colder areas globally for proliferation of the bacterium. Additionally, we address potential measures that can help mitigate the impact of climate change on *V*. *vulnificus* disease burden. Finally, we provide a blueprint, whereby understanding global warming through the lens of specific agents can help elucidate the mechanisms by which climate change influences infectious disease dynamics of other pathogens.

## Climatic disasters fuel disease outbreaks in endemic regions

One of the most significant consequences of climate change has been its impact on extreme weather events. For example, rising sea-surface temperatures (SST) have increased the incidence and intensity of hydrologic disasters like hurricanes, marine heatwaves, and floods, over the last 4 decades, disrupting both public health and global economies alike [2,3]. Its impact on human health is especially pronounced along tropical coastlines, which serve as hotspots for a plethora of native climate-sensitive pathogens or their vectors including Dengue [2], *Staphylococcus aureus* [41], and *Vibrio cholerae* [42], among others. Such weather disturbances eliminate physical barriers separating these infectious agents from their hosts, thus providing a suitable avenue for disease outbreaks (**Fig 1B**). One such example is the southeastern state of Florida in the US, which, due to its location and warm climate, experiences major tropical storms (sustained winds >110 mph associated with heavy rainfall) annually [43]. Concerningly, an increasing number of these storms have been associated with post-disaster outbreaks of *V*. *vulnificus*, a pathogen native to the aquatic ecosystems of the state. Concomitantly, Florida has become an epicenter for *V*. *vulnificus* infections, recording the highest number of cases and deaths in the US each year [44]. Furthermore, we recently showed that *V*. *vulnificus* strains with pathogenic potential, e.g., those capable of causing sepsis in animal models, naturally circulate within its coastal ecosystems [45]. These factors make *V*. *vulnificus* in Florida a suitable candidate to investigate the relationship between climatic disasters and autochthonous pathogens.

### Influence of climatic disasters on *V*. *vulnificus* outbreaks

**Fig 2:**
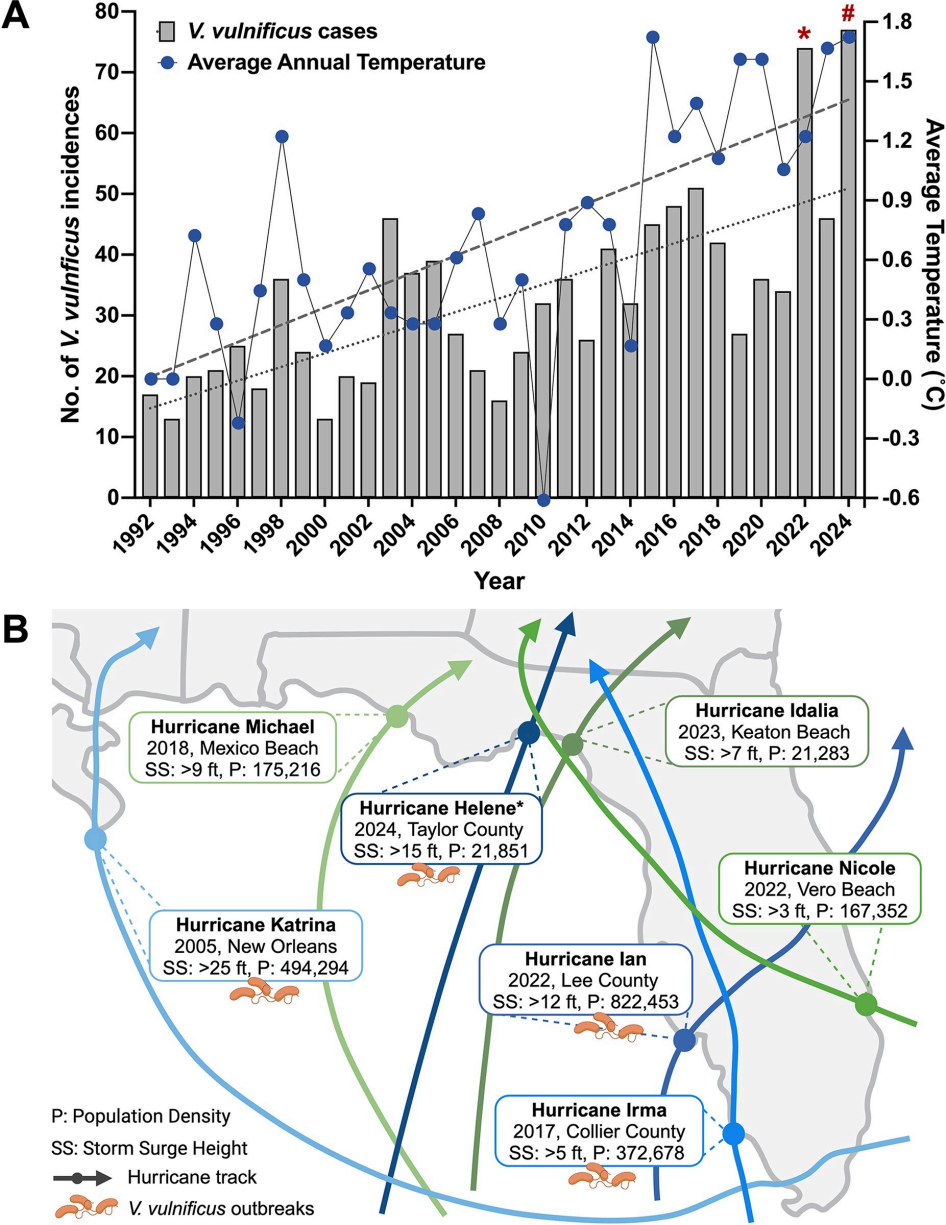
*V*. *vulnificus* disease burden in Florida. (**A**) Relationship between *V*. *vulnificus* cases (gray bars; left y-axis) and average annual temperatures (blue circles; right y-axis) in Florida. Annual *V*. *vulnificus* case counts up to October 2024 were retrieved from the Florida DoH Reportable Disease Frequency Report. Average annual temperatures (°C) were retrieved from the *Climate at a Glance*, *Statewide Time Series* database up to August 2024, and temperatures relative to the 1992 mean were plotted. Trendlines for *V*. *vulnificus* cases (dotted line) and average temperatures (dashed line) were generated using simple linear regression. Significant increase in *V*. *vulnificus* cases (+1.130 cases/year; *p* < 0.0001) and average temperatures (+0.0437°C/year; *p* < 0.0001) were observed. Correlation between the 2 variables, measured using Pearson R Correlation analysis, was found to be positive and significant (r = 0.59; *p* < 0.001). *Indicates abnormal increase in cases due to Hurricane Ian. ^#^Indicates abnormal increase in cases due to Hurricane Helene. (**B**) Storm paths of hurricanes that recently impacted Florida and the Gulf of Mexico. Those that resulted in *V*. *vulnificus* outbreaks are colored in blue. Year and region of impact, storm surge height (SS), and population density (P) are indicated. Hurricane tracks were obtained from National Weather Service database and demographic information from the US Census Bureau. *Indicates cases observed outside the region of landfall. Created in BioRender. Almagro-Moreno, S. (2024) https://BioRender.com/w21e424.

According to the Florida Department of Health (DoH), *V*. *vulnificus* disease incidences have increased significantly from 1992 to 2024 in Florida (**Fig 2A**) [46]. These case counts positively correlate with the rate at which the local SST has increased during the same period (+0.044°C/year) [47]. Interestingly, in 2022, Florida recorded an abnormal surge in *V*. *vulnificus* infections with a total of 74 cases and 17 deaths (**Fig 2A**) [39,46,48]. The spike in cases is credited to one of the deadliest hurricanes ever to strike Florida, Hurricane Ian, a category 4 storm that made landfall in Lee County on 28th September 2022 (**Fig 2B**) [49]. Hurricane Ian caused storm surges that reached heights of 12 to 18 ft in many coastal areas, leading to severe flooding and destruction [43]. This was promptly followed by the largest outbreak of *V*. *vulnificus* in US history [49]. With more than 50% of cases in 2022 (38 cases, 11 deaths) occurring during the week following the storm, these numbers far exceeded the predicted 3 cases for that period in Lee County [22,39,46]. However, the *V*. *vulnificus* outbreak after Hurricane Ian was not an isolated occurrence. More recently, on 26th September 2024, Florida experienced Hurricane Helene, which made landfall as a category 4 storm in Taylor County, Florida [50]. The unusually large diameter and significant storm surge of Helene triggered another wave of *V*. *vulnificus* outbreaks in several regions outside of the Big Bend [46,50]. With 77 cases and 15 deaths as of October 2024, and case counts still rising, this represents the highest ever recorded *V*. *vulnificus* incidences in a single year in Florida [46]. Similarly, Hurricane Katrina made landfall in Louisiana as a category 3 storm in August 2005 (**Fig 2B**) [51]. The heavy rainfall and flooding led to several clusters of *V*. *vulnificus* outbreaks (15 cases, 3 deaths) in affected areas, including Florida, within the following week [52]. Likewise, in 2017, Hurricane Irma led to 6 storm-related vibriosis cases in Collier County, Florida [39], the highest in this region prior to 2022 (**Fig 2B**) [46]. Regardless of the magnitude, each of these storms had similar outcomes: an abrupt spike in *V*. *vulnificus* infections compared to projected numbers. Based on these trends, the threat of rapid surges in cases following climatic disasters appears to outweigh the health risk posed by a gradual rise in cases over time in these vibrio-native areas. Nonetheless, not all climatic anomalies are associated with *V*. *vulnificus* outbreaks. For example, Hurricanes Michael (2018), Nicole (2022), and Idalia (2023) did not trigger subsequent *V*. *vulnificus* cases (**Fig 2B**), although storm surges were reported in regions where pathogenic strains of *V*. *vulnificus* are known to be present [45,53,54]. To elucidate these disparities, we discuss potential risk factors that contribute to differential post-disaster outcomes in endemic areas (**Table 1**).

**Table 1:** Factors driving *V*. *vulnificus* disease dynamics.

Region	Risk factors/ecological drivers	Effect	Other pathogens
Endemic	High population density	Increase in coastal populations amplify number of exposed individuals	*C*. *tetani*, *P*. *aeruginosa* [105]
	Host susceptibility	Individuals with preexisting health conditions are at higher risk of infections	*Leptospira* spp., *Aspergillus* spp. [114,115]
	Lack of disaster preparation and management	Delayed evacuation of at-risk individuals; limited public awareness and control measures	*Leptospira* spp., *Enterococcus* spp. [107,116]
	Favorable ecological niches	Environments where favorable abiotic and biotic conditions increase prevalence of pathogenic strains	*A*. *hydrophilia*, *Penicillium* spp. [106,117]
Non-endemic	Increased temperature	Abundance and environmental persistence; activation of virulence traits	Influenza virus, *Salmonella* spp. [5,108]
	Alterations in weather conditions	High rainfall and melting of glaciers increase suitability of non-endemic areas	*Plasmodium* spp., *Haemophilus influenzae* [110]
	Presence of biotic communities	Natural reservoirs provide shelter, nutrients and activate virulence factors	*V*. *cholerae*, *V*. *parahaemolyticus* [111,112]
	Increased prevalence of vectors	Vehicles of transmission increase spread and virulence	Norovirus, *Borrelia burgdorferi* [118,119]

### Factors associated with disaster-driven outbreaks

*a) Population density*. Nearly 40% of the US population lives along coastlines, with Florida having the fifth highest coastal population (approximately 15.8 million) [55]. Hurricane Ian impacted the Cape Coral–Fort Myers area which had an estimated resident population of 822,453 in 2022 (**Fig 2B**) [56]. Similarly, in 2005, the impact of Hurricane Katrina extended from Miami-Dade County, Florida, to New Orleans, Louisiana, spanning multiple populated regions along the coast, as did Hurricane Irma in the populous Collier County, Florida (**Fig 2B**) [56]. Furthermore, increased summer tourism to these popular destinations, which corresponds with the hurricane season, further exacerbates the number of individuals that can be exposed [57,58]. Contrarily, the lower population densities at Keaton and Vero beaches (**Fig 2B**), areas where hurricanes Idalia and Nicole made landfall, respectively, likely contributed to the lack of post-hurricane *V*. *vulnificus* case reports [56]. Interestingly, although Hurricane Helene made landfall in the same sparsely populated area as Hurricanes Idalia (2023) and Michael (2018), *V*. *vulnificus* outbreaks were reported in multiple counties outside this region. The substantial reach of Helene (approximately 420 miles wide) appears to have extended the storm damage as far south as the Tampa Bay region with over 3 million residents [56], where most of the post-hurricane cases were reported [46]. Heavily populated areas, therefore, have a higher probability of disease outcomes due to a greater number of individuals being exposed to the bacterium already present in the storm-surge waters.

*b) Coastal demography and host susceptibility*. Epidemiological surveillance data indicate that age increases susceptibility to *V*. *vulnificus* infections (**Table 1**) [59,60]. Specifically, individuals over the age of 45 are at higher risk of contracting the disease [59,61]. Furthermore, other risk groups include males, immunocompromised individuals, and patients with liver cirrhosis, hepatitis B or C, diabetes, or hemochromatosis [61,62]. Florida, a popular retirement destination, has drawn significant immigration of individuals over the age of 65, particularly along shorelines and riverbanks [55,63]. This is indicated by an 89% increase in their demography from 1970 to 2010 in coastal counties due to migration [55]. Consequently, such individuals are at a greater risk of disease outbreaks fuelled by hydrologic disasters. Accordingly, more than 92% of *V*. *vulnificus* infections following Hurricane Ian were reported in individuals older than 65 years [46]. Therefore, increase in susceptible demographics along coastlines amplifies the risk of *V*. *vulnificus* disease transmission in already populated disaster-prone regions.

*c) Disaster management and response time*. The National Weather Service issues public advisories to potential zones of impact well in advance of weather emergencies. This allows local authorities to take appropriate actions to ensure safety of their residents, especially the elderly and those with special needs. During Hurricane Ian, repeated Watch Advisories for severe storm surges and hurricane force winds were delivered to multiple high-risk coastal areas 36 to 48 h ahead of the storm [64]. Yet, Lee County residents were not ordered to evacuate until after a Hurricane Warning was issued approximately 24 h prior to its landfall (**Table 1**) [65]. However, the estimated time for safe evacuation from maximum risk zones in Lee County is 36 h [66]. Loss of valuable time likely left countless individuals stranded and vulnerable to impacts of the storm. As a result, many more susceptible individuals were exposed to contaminated floodwaters, likely contributing to the unprecedented surge in *V*. *vulnificus* infections post-hurricane. Lessons from this storm highlight the importance of timely response to approaching disasters and appropriate preparation programs.

*d) Proximity to favorable environments*. *V*. *vulnificus* thrives in environments where mixing of marine and fresh water provides nutrients and salinities favorable for its growth and proliferation [26,35,45]. When hurricanes approach coastlines near estuaries, the resultant wind gusts and heavy rainfall intensify the mixing of ocean and freshwater, increase nutrient loads by sediment resuspension, and dilute salinity to <25 ppt [22,67]. These changes facilitate *V*. *vulnificus* growth and, as waters containing the bacterium flood populated areas, increase exposure of susceptible individuals to the pathogen, causing potential outbreaks. When Hurricane Ian made landfall near the Caloosahatchee Estuary, Florida, these factors likely contributed to the subsequent *V*. *vulnificus* outbreak (**Fig 1B**) [22,39,49]. In contrast, neither hurricane Nicole nor Idalia struck estuaries close to populated regions. Lack of a suitable niche for proliferation of the bacterium could have contributed to the absence of storm-driven *V*. *vulnificus* infections in these areas [53,54]. Furthermore, disasters like hurricanes can weaken or destroy infrastructures that treat wastewater or maintain quality of drinking water [68]. Consequently, discharge of polluted water, rich in organic content, into the environment or drinking water supply can lead to alterations in microbial communities, including increased abundance and proliferation of *V*. *vulnificus* [68]. Thus, proximity of climatic disasters to ecosystems favorable for proliferation of *V*. *vulnificus* populations exacerbates disease risk, particularly in regions with preexisting deficiencies in water infrastructures.

*e) Prevalence of pathogenic strains*. Although *V*. *vulnificus* is ubiquitous in brackish waters, not all strains can cause disease outcomes, and virulent strains of the bacterium have been found associated with specific ecological niches [45]. For instance, a recent study from our group compared prevalence of *V*. *vulnificus* in ecologically distinct sites along a large estuary in eastern Florida, the Indian River Lagoon (IRL) [45]. Strains with pathogenic potential, i.e., able to evade the bactericidal effect of serum, resist phagocytosis by human macrophages, and cause sepsis in an animal model, were enriched in areas where salinity levels were favorable (approximately 25 ppt) and algal content was elevated [45]. Incidentally, these regions were also reported to have the highest *V*. *vulnificus* case counts in Eastern Florida within the study duration [46]. Conversely, in sites lacking favorable biotic and abiotic patterns, potentially pathogenic strains were virtually absent from the environment [45], and no cases were reported in these areas [46]. Furthermore, high chlorophyll levels in the water after Hurricane Ian, indicative of increased algal content, were concomitant with the *V*. *vulnificus* outbreak in its aftermath [22]. In addition, alterations in meteorological variables, including increase in water and air temperatures or decrease in mean wind speed and sea-level pressure, were found to be significantly correlated with increasing incidence of *V*. *vulnificus* infections [69]. These observations were corroborated in a recent study employing satellite remote sensing to identify changing environmental parameters linked to vibriosis [70]. The study concluded that the likelihood of *V*. *vulnificus* infections is significantly influenced by elevated SSTs and chlorophyll concentrations during the preceding months. These combined results indicate that specific environmental factors appear to be critical proxies for determining the likelihood of pathogenic strains of *V*. *vulnificus* thriving in an area. Thus, it is important that surveillance platforms incorporate these factors to accurately forecast the risk of disease outbreaks.

### Re-evaluation of control measures in endemic areas

The aforementioned factors indicate that risk prediction models for disaster-driven *V*. *vulnificu*s outbreaks can be developed effectively (**Table 1**). For instance, when climatic disasters impact estuaries harboring pathogenic *V*. *vulnificus* strains, the resulting heightened proliferation of the bacterium poses a significant risk of disease outbreaks in coastal areas with high population densities that include vulnerable communities. Concerningly, climate change is projected to increase the frequency, duration, and intensity of hurricanes, prompting researchers to propose extending the current Saffir–Simpson Hurricane Wind Scale to include a Category 6 for exceptionally intense storms [71]. Aggravation of such disasters will, in turn, elevate risk of disease outbreaks in such communities. Therefore, it is critical that local authorities in high-risk regions amend disaster preparation programs to include prevention of *V*. *vulnificus* outbreaks. Namely, precautionary measures against the bacterium must be included in hurricane preparations, such as recognizing infection symptoms, administering first aid, and increasing awareness to avoid exposure to the storm waters. Furthermore, re-evaluation of disaster management strategies must be considered to allow for longer evacuation times, especially for elderly and special needs residents. In addition, regular water monitoring for presence of pathogenic *V*. *vulnificus* strains and environmental parameters conducive to their proliferation in disaster-prone regions is vital. The majority of environmental *V*. *vulnificus* monitoring to date has been routinely conducted in developed countries, namely US and Europe, and the lack of *V*. *vulnificus* surveillance in low-income countries is a concern. These strategies will thus help determine the potential risk of post-disaster *V*. *vulnificus* outbreaks globally and aid in development of appropriate control measures.

## Climatic suitability drives geographical expansion of infectious diseases

**Fig 3:**
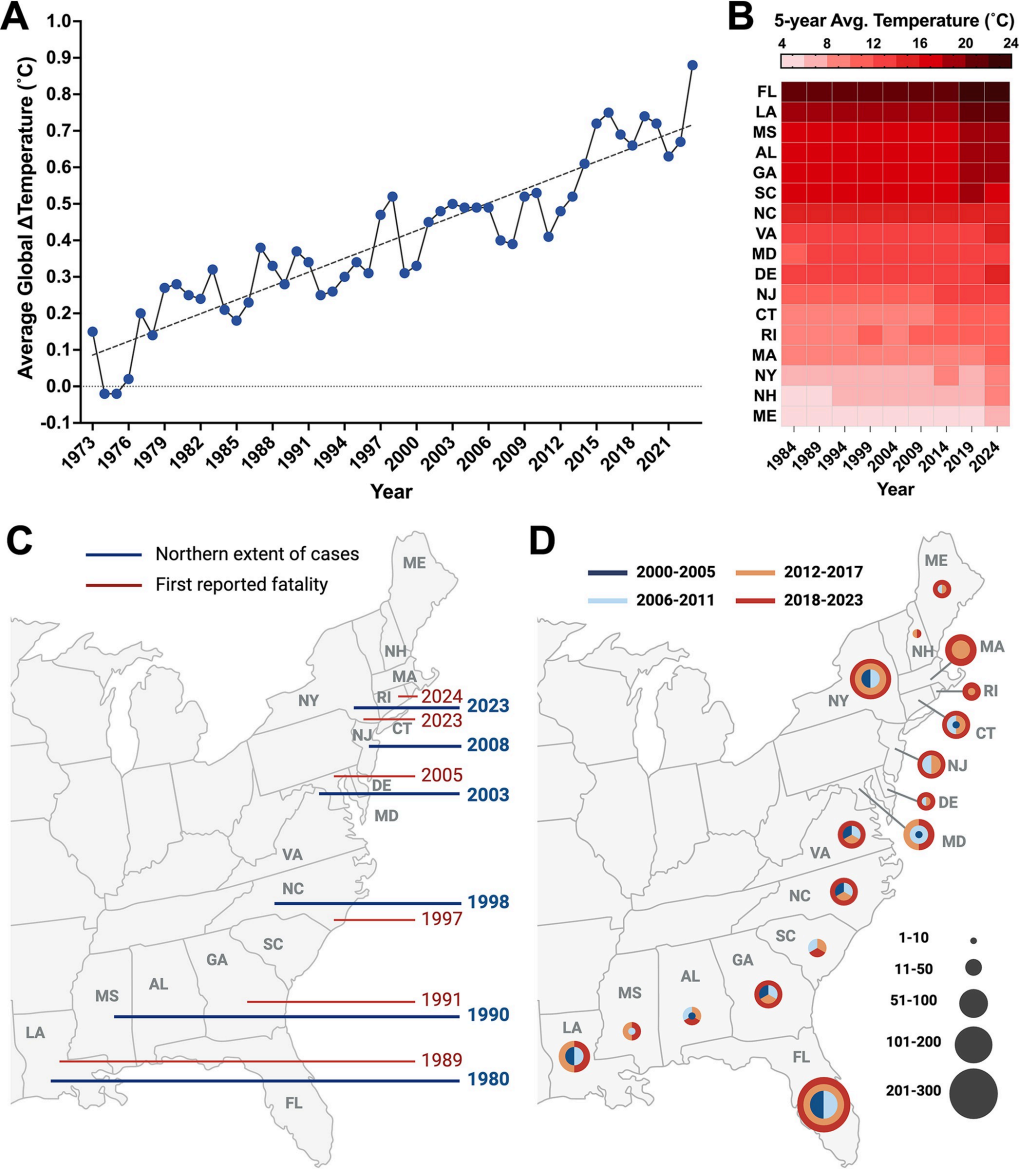
Spatiotemporal distribution of pathogenic *Vibrio* spp. (**A**) Annual average global temperatures relative to the 19th century (1901–2000) average. Trendline (dashed line) was generated using simple linear regression and indicates a significant increase in the temperature (+0.013°C/year; *p* < 0.0001) over 50 years. Temperature data were retrieved from *Climate at a Glance*, *Global Time Series* in the National Center for Environmental Information database. (**B**) Temporal trends in average annual temperature along the Gulf and Atlantic Coasts of the US Heat map represents five-year averages of annual temperatures (°C) per state for the following time periods: 1981–1984, 1985–1989, 1990–1994, 1995–1999, 2000–2004, 2005–2009, 2010–2014, 2015–2019, 2020–2024. X-axis indicates the fifth year for each time period. Annual temperatures per state were retrieved from the *Climate at a Glance*, *Statewide Time Series* database up to August 2024. (**C**) Geographical distribution of *V*. *vulnificus* cases across Eastern USA. The northern extent of *V*. *vulnificus* infections from non-foodborne sources has expanded from latitude 32°N in 1990 to >41°N by 2023. Blue lines indicate year of the northernmost *V*. *vulnificus* cases and red lines first reported fatalities. Northern extent of cases were adapted and modified from Archer and colleagues [17] and case fatalities were retrieved from data reported to the CDC and regional health departments. Created in BioRender. Almagro-Moreno, S. (2024) https://BioRender.com/u42n067. (**D**) Geographical distribution of Vibriosis cases (caused by any species of the *Vibrionaceae* family, other than toxigenic *V*. *cholerae* O1 or O139) in Eastern USA. Annual number of Vibriosis cases per state was retrieved from the CDC Vibrio Surveillance System (2000–2018), Cholera and Other Vibrio Illness Surveillance (COVIS) database (2009–2015), and WONDER Annual Tables of Infectious Diseases and Conditions database (2016–2023). For each state, average Vibriosis cases per 6-year bracket (dark blue, 2000–2005; light blue, 2006–2011; orange, 2012–2017; red, 2018–2023) are depicted as circles, with increasing radii indicating increase in average cases. Year brackets where change in average cases remains within a specified range are represented as parts of a circle. If the average number of Vibriosis cases for 2 consecutive years within a 6-year bracket was ≤1, the average for that bracket was considered as 0 and not represented on the map. Created in BioRender. Almagro-Moreno, S. (2024) https://BioRender.com/m68j476.

Alterations in weather patterns have been driving the spatiotemporal expansion of several infectious diseases to areas with no prior reports of their incidences [5,6,8]. SSTs have been rising worldwide for the past 50 years at an average rate of 0.013°C per year (**Fig 3A**), with 2023 recording the highest temperature in over 174 years (+0.91°C from 19th century mean) [47,72]. Such temperature anomalies and subsequent changes in rainfall and humidity patterns have expanded globally. Consequently, the extent of regions becoming ecologically suitable for the proliferation of water-, food-, and vector-borne pathogens is expanding [2–4]. This has led to increased incidences of diseases such as salmonellosis, dengue, chikungunya, and leptospirosis in regions where they were previously uncommon (**Fig 1A**) [4,8,10]. Recent projections predict that, with rising SSTs, transmission periods and vector breeding sites for these pathogens will significantly increase within the next decade, worsening associated disease outcomes, especially among coastal populations [2,72]. One such emerging threat is the expansion of *V*. *vulnificus* infections to regions with no history of reported cases [17,20,21]. This has been attributed to the increased extent of coastlines suitable for its proliferation, measured based on changes in salinity and SST [2,73]. The sensitivity of *V*. *vulnificus* to global warming and related environmental changes, the rapid and high mortality rate of its infections, and its heavy economic burden, warrants the urgent need to address this expanding geographical threat. Below, we discuss recent trends in the spatiotemporal expansion of *V*. *vulnificus* infections and review key drivers increasing the environmental persistence and pathogenic capabilities of this bacterium in colder latitudes.

### Influence of climate change on the spatiotemporal distribution of *V*. *vulnificus* infections

In eastern US, *V*. *vulnificus* infections have increased 8-fold between 1988 and 2018 [59]. This is further compounded by a northward shift of its geographical case limit by *ca*. 48 km/year [59] (**Fig 3B**), encompassing latitudes where such infections were previously uncommon [17]. For instance, while *V*. *vulnificus* infections rarely occurred in locations northward of Georgia in late 1980s (32°N), cases were reported as far north as Philadelphia by 2018 (40°N) (**Fig 3B**) [17]. Consistent with this trend, in summer 2023, 12 *V*. *vulnificus* related deaths were reported in major population centers of New York and Connecticut, regions where infections were virtually nonexistent until recently [37]. This shift in cases corresponds with a 27% increase in extent of the US northeastern coastline favorable for *Vibrio* spp. (**Fig 3C**) [73]. These unprecedented changes led the Center for Disease Control and Prevention to issue an emergency health advisory against “severe *V*. *vulnificus* infections in the US associated with warming coastal waters” [37]. Concerningly, if current trends and limited actions against global warming continue, *V*. *vulnificus* cases are predicted to expand into Massachusetts, New Hampshire, and Southern Maine by 2041 to 2060 [17]. Surprisingly, in contrast to the Atlantic coast, the Pacific US coast has not experienced a comparable rate of increase in *V*. *vulnificus* cases [59,60,74]. This is likely due to the overall higher salinity levels (30 to 40 ppt) along the west coast [74] which does not support long-term prevalence of *V*. *vulnificus*. In addition, the SST along the Pacific coast is generally much lower than that of the Atlantic coast, with monthly averages during the summer ranging from 13°C to 16°C [75], well below the optimal conditions for *V*. *vulnificus* proliferation. Nonetheless, parts of the Pacific are warming at an accelerated rate, reaching record-high temperatures (>20°C) in 2023 in many regions due to global warming [75,76]. Given such changing weather patterns and the high recreational and commercial water use in these parts, such coastal communities are at a heightened risk for future *V*. *vulnificus* outbreaks.

A similar longitudinal increase in *V*. *vulnificus* infections is occurring globally. For instance, the Baltic Sea, one of the largest brackish aquatic ecosystems worldwide, harbors native populations of *V*. *vulnificus* along its southern coastal belt [20]. However, since 2014, extreme heatwaves in this region have caused an expansion of *V*. *vulnificus* infections to several sub-arctic regions of Northern Europe [34,77]. By 2018, cases had expanded as far north as Finland (latitude >65°N), nearly 160 km from the Arctic Circle, the highest northern latitude where *V*. *vulnificus* infections have been reported [16,34]. Furthermore, the total coastal area suitable for growth and proliferation of pathogenic *Vibrio* spp. has increased by 392 km^2^ since 1982 [2,73]. This includes a record 81 countries that now have conditions favorable for *V*. *vulnificus*, including 17% of the European and Asian coastlines [2]. Interestingly, *V*. *vulnificus* cases are relatively infrequent in South America, which is surprising given the bacterium has been isolated from water and seafood samples in these parts [78]. Similar to the Pacific US coast, these regions also have generally higher salinity levels (>30 ppt) [78] and likely do not support proliferation of pathogenic *V*. *vulnificus* [45]. Additionally, limited surveillance, inconsistencies in data collection, and underreporting of *Vibrio* infections across South America likely contribute to the overall low case counts [78]. Nonetheless, this trend might be short-lived as *V*. *vulnificus* cases have increased along the Atlantic coast since 1997, with several new outbreaks reported as far south as Uruguay during the past 15 years [78,79].

In addition to the expansion of favorable ecological niches, increased human population densities along the coast of many countries further aggravate the risk of *V*. *vulnificus* infections. The global population within 100 km of coastlines reached more than 1.4 billion in 2022. Asia and Europe have the largest populations within 1 km of the coast and as discussed above, these areas are experiencing a rapid expansion in *V*. *vulnificus* cases. At the current rate, cases are expected to increase by 23% to 39% even if the increase in SST is limited to 2°C by mid-century [2]. However, with no action taken, the extent of coastlines suitable for rapid growth and proliferation of *V*. *vulnificus* will increase by 64% to 84% globally, with number of cases amplifying by 102% to 140% [2]. It is therefore vital that we understand climate-driven environmental changes that amplify natural *V*. *vulnificus* populations in the aquatic ecosystem and increase their disease transmission potential in regions where cases are presently uncommon. Below we discuss key drivers that facilitate the expansion of *V*. *vulnificus* populations and highlight their role in increasing its infectivity (**Table 1**).

### Environmental parameters driving *V*. *vulnificus* disease expansion

*a) Temperature*. Several lines of evidence provide strong correlation between rising SST and *V*. *vulnificus* cases (**Fig 1A**) [20,23,34], typically attributed to increased abundance of the bacterium. For instance, bacteriological surveillance of environmental samples collected in Chesapeake Bay identified 2 critical thresholds for abundance of culturable *V*. *vulnificus*: an initial increase in detectable numbers at 15°C and a second increase associated with maximum counts at 25°C [35]. Nonetheless, the role of temperature in virulence and disease transmission of *V*. *vulnificus* has been scantily addressed [21,35]. One study demonstrated that temperatures >25°C activated adaptive traits in *V*. *vulnificus* that facilitate host colonization including motility and chemotaxis, protease activity, stress resistance (antimicrobial compounds, oxidative stress), and metabolism (amino acids, iron, fatty acids, lipids) [80]. Rising SST may, therefore, pre-adapt potentially pathogenic strains for infections or may be a selective pressure for virulent clones of the bacterium. In addition, temperatures >20°C delay entry of *V*. *vulnificus* cells into a viable but non-culturable state and trigger resuscitation from this state [81,82]. Prolonged summers due to climate change will thus allow the bacterium to remain proliferating for longer durations, extending its transmission period. Extended warm periods also attract increased recreational activities and commercial fishing thus increasing the frequency of exposure to the pathogen. Therefore, an increase in SST not only expands areas suitable for *V*. *vulnificus* proliferation, but also increases its environmental persistence and activates key virulence traits, thus accelerating risk of disease outcomes.

*b) Salinity and plankton*. Aside from SST, salinity and phytoplankton composition appear to be accurate predictors of *V*. *vulnificus* density and virulence [35,45]. Nonetheless, climate-driven weather changes are modulating salinity and phytoplankton levels in areas non-endemic to *V*. *vulnificus* disease (**Fig 1A**). For instance, increased rainfall, which amplifies influx of freshwater into the ocean, and melting of polar ice caps, has reduced ocean salinity to moderate levels (approximately 25 ppt) [67,83]. Increasing availability of such favorable estuarine environments would thus provide a suitable avenue for its enhanced proliferation, which corresponds with higher rates of disease incidences. A significant occurrence of climate-driven decreasing ocean salinity is observed in the North Atlantic subpolar gyre between latitudes 45°N and 70°N [84,85], regions that coincide with the expanding range of *V*. *vulnificus* infections [17]. Furthermore, abundance of phytoplankton species that serve as nutrient-rich natural reservoirs and indicators for *V*. *vulnificus* has expanded worldwide over the last 3 decades [86,87]. This expansion is attributed to elevated SSTs, adequate salinity, increased eutrophication and rainfall, resulting in harmful algal blooms [88–92]. In addition, *V*. *vulnificus* is commensal with zooplankton, like copepods, which provide nutrient-rich surfaces for bacterial attachment [15,93]. Since zooplankton feed on phytoplankton, correlation of zooplankton population sizes following phytoplankton blooms can be calculated [15,93]. Therefore, conditions favorable for expansion of zooplankton are also associated with an increase in abundance of *V*. *vulnificus* [15,40]. With the onset of climate change, these abiotic and biotic alterations will continue to spread globally, increasing the risk of disease incidences in non-endemic areas.

*c) Mollusks*. Filter-feeding mollusks, e.g., oysters and clams, are primary vehicles of transmission for food-borne *V*. *vulnificus* infections [94,95]. By filtering large amounts of surrounding water, these bivalves concentrate bacteria by up to 6 × 10^4^ CFU/gram of tissue [96]. Interestingly, presence of surface virulence factors, such as capsular polysaccharide, pili, and flagella, were found to be essential for the uptake of *V*. *vulnificus* into oysters [97]. Furthermore, changes in climatic factors like elevated temperatures (>15°C) and favorable salinity (5 to 25 ppt) have been shown to increase uptake and prevalence of *V*. *vulnificus* in the oyster microbiome [98,99]. Nonetheless, while mechanisms of these interactions remain to be fully elucidated, such studies further highlight the numerous ways in which environmental reservoirs can serve as selective pressures for this pathogen.

### Limiting the public health risk of disease expansion

Numerous lines of evidence point toward complex and nonlinear relationships between and among parameters strongly influenced by climate change, such as abiotic factors described above (SST, salinity), natural reservoirs (phytoplankton, oysters, zooplankton), and the abundance and infectivity of *V*. *vulnificus*. With global warming reaching unprecedented heights, the convergence of these factors has increased the public health risk of *V*. *vulnificus* infections worldwide (**Table 1**). Therefore, incorporating both biotic and abiotic parameters into predictive models will help forecast at-risk regions where *V*. *vulnificus* outbreaks are expected to occur. Developments in remote sensing have allowed access to near real-time environmental and sociological data, enabling predictive models to assess risk of *Vibrio* infections. A successful example of such an undertaking is the cholera predictive model implemented in Yemen which, coupled with interventions early in outbreaks, has enabled the prediction of cholera risk at least 4 weeks in advance [14,100]. Similar models have been developed for *V*. *vulnificus* in the Chesapeake Bay [101]. Nonetheless, predictive modeling relies on ground observations to train algorithms, necessitating additional ground surveillance of non-cholera *Vibrio* spp. before such models can be applied on a global scale. Such platforms will enable continuous monitoring of conditions that increase *V*. *vulnificus* abundance and virulence, and where appropriate disease management efforts can be implemented. Understanding how ecological factors drive the virulence of *V*. *vulnificus* or select for pathogenic variants will allow identification of potential sources of outbreaks. Lastly, measures that limit the spread of algal blooms, notably reducing nutrient loading and improving the design of sewage networks, will be essential to prevent enhanced proliferation and potential disease transmission of this pathogen globally [102].

## Conclusions

Climate-related factors influencing transmission of infectious diseases are beginning to be elucidated. Nonetheless, it is becoming abundantly clear that the relationship between climatic factors and infectious agents involve a network of nonlinear interactions. The complex interplay between disease-causing agents and their natural ecosystems makes it difficult to isolate the individual contributions of such factors in driving the emergence and spread of infectious diseases. Nonetheless, understanding these relationships and their synergistic effects on disease dynamics represents one of the most difficult contemporary public health challenges. In this review, we unveil trends that simplify the multilayer relationship between risk factors and infectious agents by consolidating climatic pathways into 2 broad categories: those affecting regions endemic or non-endemic to infectious diseases. This enabled the identification of several risk factors fueling *V*. *vulnificus* outbreaks (**Table 1**) which can be broadly applicable to numerous other climate-sensitive pathogens under both scenarios. Therefore, mitigation efforts aimed at controlling one disease would also naturally contribute to the control of others.

In disaster-prone regions native to climate-sensitive pathogens, risk factors driving post-hurricane *V*. *vulnificus* outbreaks (**Table 1**) appear to influence incidences of several other infectious diseases following climatic disasters [41,42,103,104]. For instance, 2004 the Indian Ocean tsunami significantly affected multiple densely populated coastal communities likely serving as a primary catalyst for the subsequent outbreaks of *Pseudomonas aeruginosa*, *Klebsiella pneumoniae*, and *C*. *tetani* [105]. Additionally, following Hurricane Katrina, high concentrations of the pathogen *Aeromonas hydrophilia* were detected in floodwaters near New Orleans [106]. Proximity of the hurricane to Lake Pontchartrain, an estuarine system with favorable post-storm ecological conditions likely exacerbated proliferation of this pathogen [106], similar to the impact of Hurricane Ian on *V*. *vulnificus*. Furthermore, when Hurricane Maria made landfall in Puerto Rico, preexisting deficiencies in healthcare and water infrastructures and inefficient relief management efforts resulted in an outbreak of leptospirosis [107]. Addressing these common drivers (**Table 1**) would thus enhance prediction, prevention, and mitigation efforts, enabling public health interventions and improved preparedness in vulnerable regions. For instance, strengthening infrastructure for safe drinking water, sanitation, and hygiene in densely populated areas could help prevent the spread of waterborne diseases like cholera, typhoid fever, and dysentery. Pre-positioning mobile medical units in strategic locations, such as closer to evacuation centers which typically observe spikes in multiple infectious diseases [41] could ensure quicker access post-disaster healthcare. Conducting pre-disaster public health campaigns on hygiene, disease prevention, and vaccination could help prepare communities for potential outbreaks after a disaster.

Similarly, in non-endemic regions, alterations in environmental parameters such as temperature, rainfall, and salinity, have been linked to the spread of multiple climate-sensitive pathogens (**Table 1**). For instance, higher temperatures are increasing the spread of viruses (COVID-19, influenza), enteric bacteria (*Shigella*, *Salmonella* spp.), or disease-carrying vectors (*Anopheles*, *Aedes* mosquitos) globally [2,5,108]. Besides, ocean warming driven rising sea levels has caused the expansion of brackish water bodies in coastal zones [109]. The resultant increases in salinity favor transmission of salinity-tolerant mosquitoes that serve as vectors of diseases such as malaria and dengue [109]. On the other hand, heavy rainfall, which reduces seawater salinity levels, has been linked to increased incidences of diarrhea, pneumonia, and enteric fever [110]. Furthermore, planktonic communities, which are expanding globally due to climate change [88–92], serve not only as environmental reservoirs for several pathogenic *Vibrio* spp., but also modulate their virulence likely facilitating their spread and infectivity [111,112]. Understanding the commonalities between and among the ecological drivers of infectious diseases can lead to control strategies that target multiple pathogens simultaneously, optimizing resource allocation and maximizing public health outcomes. This would also help design effective surveillance programs that monitor not just individual diseases but also the ecological context in which they occur, enhancing early detection and management of outbreaks.

Importantly, the critical role of societal dynamics in climate-driven disease distribution and incidence has been recently gaining importance. These social factors include economic growth, changes in population and demographics, urbanization, regulatory policies, and political engagement in science and climate change. Limited studies have incorporated such factors in predictive models and demonstrated improved effectiveness in projecting future trends of disease distribution [2,17,113]. However, integration of social parameters into predictive risk modeling is far from being widely adapted and implemented. Given the plethora of socioeconomic and political factors involved at the regional, national, and global levels, consistent data collection is critical towards improving the accuracy and predictive power of disease models. Furthermore, the sheer number and range of diseases influenced by climate change makes it imperative that an international database of such diseases be developed and accessible [5,44,113].

Overall, the vast amalgamation of interactions between climatic factors and pathogens via socioeconomic or ecological influences makes it particularly challenging to develop effective countermeasures [5]. We contend that systematic approaches like the one presented here, which aligns with the One Health approach of recognizing the interconnectedness between such factors, will help disentangle these complex networks. Although the specific climatic determinants that influence disease dynamics may vary across pathogens, the risk factors addressed above, and the measures proposed in this review will be broadly applicable to several climate-sensitive pathogens. Ultimately, this would help create avenues that lead to the development of feasible plans to ensure global public health safety in the face of this global threat.
